# Comparative Genomic Analysis of *Mycobacterium tuberculosis* Isolates Circulating in North Santander, Colombia

**DOI:** 10.3390/tropicalmed9090197

**Published:** 2024-08-28

**Authors:** Diana Patricia Bohada-Lizarazo, Karen Dayana Bravo-Sanabria, Paola Cárdenas-Malpica, Raúl Rodríguez

**Affiliations:** 1 Centro Experimental de Diagnóstico e Investigación Molecular-CEDIMOL, Universidad de Pamplona, Pamplona 503050, Colombia; karen.bravo@unipamplona.edu.co (K.D.B.-S.); rrodriguez@unipamplona.edu.co (R.R.); 2 Grupo de Investigación en Recursos Naturales, Universidad de Pamplona, Pamplona 503050, Colombia; paola.cardenas2@unipamplona.edu.co; 3 Departamento de Bacteriología y Laboratorio Clínico, Facultad de Salud, Universidad de Pamplona, Pamplona 503050, Colombia

**Keywords:** whole-genome sequencing, comparative genomics, *Mycobacterium tuberculosis*, Colombia, genotyping

## Abstract

Tuberculosis (TB) is an important infectious disease in relation to global public health and is caused species of the *Mycobacterium tuberculosis* complex (MTBC). In this study, we used whole-genome sequencing (WGS) and comparative genomics to investigate the genetic diversity of *M. tuberculosis* (*Mtb*) isolates circulating in North Santander (NS), Colombia. WGS was used for the phylogenetic and lineage characterization of 18 isolates of *Mtb* typed with orphan genotypes from 11 municipalities of NS between 2015 and 2018. The isolates studied were included in six sublineages from L4; the most frequent were 4.1.2.1, 4.3.3, and 4.3.4.2, corresponding to a proportion of 22.2%. The genome analysis conducted allowed the identification of a set of genetic variants mainly associated with determinants of virulence and evasion of the immune system (*PPE34* and *PE_PGRS2*); adaptation and survival (*PGL/p-HBAD*); stress response (*sigJ* and *sigM*); geographic variability (*PPE34*); and carbohydrate and lipid metabolism (*aldA*, *rocA*, and *cyp144*). This is the first description of the molecular epidemiology of *Mtb* isolates circulating in NS achieved through WGS. It was possible to perform comparative genomics analyses between *Mtb* isolates against the universal reference H37Rv and Colombian UT205 genome, which can help us to understand the local genetic diversity and is relevant for epidemiological studies, providing insight into TB transmission dynamics in NS.

## 1. Introduction

Tuberculosis (TB) is a public health problem that has recently increased in scale; in its last report, the World Health Organization (WHO) reported 7.5 million new cases of TB and 1.3 million deaths caused by this disease in 2022 [1], revealing it to be the infectious disease with the second-highest death toll after Coronavirus Disease 2019 (COVID-19), in addition to being the cause of twice as many deaths as Human Immunodeficiency Virus/Acquired Immunodeficiency Syndrome (HIV/AIDS) and causing around 20% of deaths among people resistant to antituberculosis drugs [2]. This disease is caused by species of the *Mycobacterium tuberculosis* complex (MTBC), which includes ten lineages (L1–L10) adapted to humans [3,4,5], in addition to the species that infect animals, which are distributed phylogeographically [6].

North Santander (NS) is a department of the Republic of Colombia with 1.7 million inhabitants located in the northeastern region and is geographically bordered by the Republic of Venezuela. In NS, the incidence of TB was 42.02 per 100,000 inhabitants in 2023 [7], a value higher than the national average rate (35.17), placing this department among those with the highest TB burden in the nation.

Regarding the mortality rate, Colombia showed relatively stable behavior in 2013–2021, with values between 2.0 and 2.2 deaths per 100,000 inhabitants; however, in 2022, it registered an increasing rate, reaching 2.6 deaths per 100,000 inhabitants [8]. In 2022, 1351 people were registered to have died from TB in this country, affecting men in a greater proportion (71.9%); the older adult group (60 years and older) registered the highest mortality rate for TB, with 10.1 per 100,000 inhabitants; at the same time, NS reported a rate of 2.5 per 100,000 inhabitants for the same year [9].

According to the Colombian study carried out in 2010–2018 [9] that estimated Disability-Adjusted Life Years (DALYs) to TB, it was found that a large part of the national territory (51.5%), including NS, has a high burden, and it results mainly from premature mortality in men [10]. NS reported 9496 cases of sick people and 814 cases of people dying from TB, with a DALY rate of 865.9 per 100,000 inhabitants, which, together with the rate of years of life lost (YLL), amounts to 774.2 cases per 100,000 inhabitants and, for years of lived with disability (YLD), 90.7 cases per 100,000 inhabitants [10]. These data are indicators that reflect the need to improve networking care and the health system in regard to the surveillance of TB events in this region of the country. All of the above information justifies the strengthening of public health policies, reducing the impact of this pathology on the population.

Genotyping has provided evidence of the genotypic diversity of MTBC isolates, such as the findings reported by Jia et al. [11], who, in a comparative analysis between 12 genomes, found large variations in the phenotypes, including regarding association with the host, virulence, and immunoreactivity, which could be the result of long-term coevolution between diverse human and animal populations [12,13,14]. Another example is the information provided by Zwyer et al. [15], who studied the numerous *Mtb* genotypes introduced anciently or recently in Dar es-Salam (Tanzania) from different regions around the world using whole-genome sequencing (WGS) and clinical data, detecting differences in transmission rates and the duration of the infectious period, while, in regard to virulence, variation was not observed between the most common circulating genotypes during an active TB infection, showing that genomes have evolved at sites that affect the transmission capacity of MTBC strains of diverse genetic origins [15].

On the other hand, a study carried out on 36 genomes belonging to lineages L4, L5, and L6 in Ghana [16] found that *Mtb* isolates from lineage 4 spread faster than those from *M. africanum* lineages, because the latter presented mutations in essential growth genes such as *ftsE*, *pstP*, *whiB3*, and *suhB*, which are associated with cell division and are responsible for cellular control [16]; furthermore, the diversity of the carbohydrate metabolic cycle was confirmed, where, in *M. africanum* lineages, evidenced a greater preference for pyruvate than for glycerol as a carbon source, similar to that for *M. bovis* [17]. The preference of L6 for pyruvate was related to modifications in the *pykA* and *eno* genes [16], demonstrating that gene variations have implications for the physiology of pathogens.

The growing use of molecular tools such as the WGS of *Mtb* and bioinformatic systems with clinical and epidemiological applications has made it easier to obtain information on genes associated with virulence [18], transmission [19], and multiplication which phenotypes are the result of mutations such as single-nucleotide polymorphisms (SNPs), i.e., smalls insertions or deletions (indels) of the different types of circulating strains [6], allowing researchers to focus on clinical and surveillance activities regarding TB in different regions of the world in order to improve the impact of the corresponding control programs.

Thus, the genomic study of *Mtb* isolates has allowed researchers to carry out comparisons using different approaches, such as searching for individual genomic variants or variants shared by all isolates through mapping against the H37Rv genome, contributing the comprehension of its evolution, adaptation to human populations, and the immune response induced by its host. For example, in a study conducted in Thailand [20], *Mtb* isolates from patients with meningeal TB and patients with pulmonary TB were compared, and it was found that 242 gene variants were common in isolates of meningeal TB and rare in isolates from pulmonary TB, highlighting 28 nonsense SNPs that affected genes such as *pks* and some of the *PE/PPE* genes, in addition to a greater number of structural variants in the isolates that cause pulmonary TB. Likewise, the authors found that the distribution of the lineages differed between the two types of disease; that is, the East Asian lineage predominated in both types, while the proportion of the Indo-Oceanic lineage was greater in the cases of meningeal TB, demonstrating some potential factors associated with the pathogenesis of meningeal TB [20,21].

In this work, we start with a genotyping study of *Mtb* isolates circulating in NS [22], where 18 isolates with an orphan genotype (based on *spoligotyping*), were taken, to which WGS and comparison were applied against the H37Rv and Colombian UT205 genomes, describing their genomic characteristics and thus determining if they have any relationship with the high transmission of TB in NS, Colombia.

## 2. Materials and Methods

### 2.1. Type of Study

This carried out a descriptive, prospective, cross-sectional study that included *Mtb* isolates stemming from cases of TB investigated and reported in the 2015–2018 period in NS, Colombia.

### 2.2. Study Population

Patients who were investigated and reported to have TB at the clinical laboratory of the ESE Hospital Universitario Erasmo Meoz (HUEM) in the city of Cúcuta, NS, Colombia, between 2015 and 2018 were included.

### 2.3. Sample Design

This study was carried out using non-probabilistic convenience sampling. The sample was formed using 215 cases of pulmonary and extrapulmonary TB; among which, 207 cultures were genotyped as *Mtb* based on *spoligotyping* and the number of tandem repeats of interspersed variable repetitive units (MIRU-VNTRs) of the mycobacteria of 24 loci. From the example, 18 isolates were taken among the 72 isolates that had an orphan genotype to perform WGS, taking into account the quality, integrity, and content of the DNA obtained from them.

### 2.4. Bacterial Culture and DNA Extraction

Initially, sputum samples were cultured in Ogawa Kudoh o Lowenstein Jensen Microgen^®^ Culture medium [23]. The samples were incubated at 37 °C, with growth observed weekly until 8 weeks, but they were not discarded as negative before 12 weeks. The cultures that showed colony growth were treated using by Ziehl Neelsen (ZN) staining to confirm the presence of Acid-Fast Bacilli (AFB), and an immunochromatographic assay (BD MGIT TBc ID, Beckton Dickinson Diagnostic, Sparks, NV, USA) was performed, validating that the isolates belonged to the MTBC. In addition, banks of both culture colony samples and genomic DNA were created, all of which were stored at −80 °C as counter samples to be used in case they were needed or in future analyses.

DNA extraction was performed on the isolates identified as belonging to the MTBC using the cetyltrimethyl ammonium bromide (CTAB) method, as previously described [24]. The amount of DNA obtained was determined using a Nanodrop 2000C Thermo Scientific^®^ (Waltham, MA, USA) with measurements at 260 nm/280 nm; calculating the equivalence to 50 µg/mL per absorbance unit, the relation 260/280 equal to 1.8 is considered the ideal ratio for pure DNA. Likewise, the quality of the extracted DNA was evaluated via electrophoresis in 0.8% agarose gel with TBE 1X buffer stained with SYBR Safe, Invitrogen (10,000×). The DNA samples that maintained integrity, purity, and quantity were subjected to sequencing [25].

### 2.5. Data Management

#### 2.5.1. Study of Variables

This study includes sociodemographic (age, sex, country and municipality of origin, and ethnicity); socioeconomic (inhabitant of a commune or township, health regime, overcrowding, and homelessness); microbiological (type of sample, smear microscopy result, culture result, and immunochromatographic test result); clinical (type of TB, comorbidities, and treatment); and epidemiological (type of patient and TB risk municipality) variables of the participants, which were taken from the notification forms of the 813-TB Event from the HUEM Clinical Laboratory and medical records.

Also, genotypic variables corresponding to the spoligo international type (SIT) of *spoligotyping*, lineages or families, and subfamilies and mycobacterial interspersed repetitive unit (MIT) of the MIRU-VNTR data were included. All this information was recorded in a Microsoft Office Excel 2016 (Microsoft Corporation—Redmond, WA, USA) spreadsheet that was developed for this purpose and used to generate the NS TB database presented in this study.

#### 2.5.2. Analysis of Variables

Initially, an exploratory analysis of the data was carried out, describing all qualitative and quantitative variable studies using absolute and percentage frequencies, as appropriate. Afterward, the grouped pattern category was compared to the single-pattern category using Fisher’s exact test for both types of variables.

Finally, for variables that presented a *p* < 0.05 in the previous analysis, the association between them and the grouped pattern was determined by calculating the prevalence ratios (PRs) with their respective confidence intervals (95% CIs) utilizing univariate Poisson regression. Additionally, a multivariate analysis was performed to determine independent variables associated with the grouped pattern. The analyses were performed using Stata 16.1 software (Stata Corp LLC, College Station, TX, USA).

### 2.6. Total Sequencing of Mtb Genomes

Sequencing was performed at the High-Throughput Genomics core of the University of Utah. DNA libraries were prepared using an Illumina DNA Prep library kit and sequenced using Illumina NextSeq (2 × 151 bp). The sequences were generated as paired ends, trimming was performed to remove adapters, and a quality analysis was run with QIIME v1.9.1 [26] and DADA3 v1.6.0 [27], revealing that all sequences exceeded a score of Q20 (Table S1).

The filtered reads were assembled with MegaHit Assembler v1.0 [28] and aligned to the reference H37Rv genome sequence (NCBI Accession: NC_000962.3) using Kraken software v2.5.0 [29] via the Python v3.12 computational language. The raw sequences were deposited in the European Nucleotide Archive under bioproject PRJEB70559 (ERP155524) and the accessions numbers ERS17287192 to ERS17287209 (available at https://www.ebi.ac.uk/ena/browser/view/PRJEB70599, accessed on 27 November 2023).

### 2.7. Annotation and Identity Analysis of Mtb Genomes

Structural annotations of the genes and other regions in the *Mtb* isolates were carried out against UT205 (GenBank accession: NC_016934.1, GI: 392384721) and H37Rv (NCBI Accession: NC_000962.3). Multiple alignment of *Mtb* genomes was performed utilizing “MAFFT Alignment” tools [30] within Geneious Prime 2023 software (https://www.geneious.com/ accessed on 10 November 2023). During this alignment process, an identity matrix and similarity was sought between the genome sequences with H37Rv and UT205 to identify the genes, regulatory regions, and other characteristic sequences. 

### 2.8. Phylogenetic Analysis

In order to evaluate the genomes behavior of interest and their homology with respect to the reference genomes, a maximum likelihood phylogenetic tree was generated with the 20 genomes using the RAxML tool v8.0 [31].

Phylogenetic analysis was performed using the general time-reversible model (GTR), which evaluates phylogenetic inferences in considerations of the reversibility of nucleotide substitutions over time. A bootstrap of 1000 resampling inferences was employed, and *M. canettii* (GenBank access: ASM25337v1) was used as an outgroup due to its divergent position and greater genetic variability in comparison with the main MTBC subspecies.

### 2.9. Identification of Mtb Variants

*Mtb* genomics variants were identified from WGS data using an algorithm available at https://github.com/ksw9/mtb-call2, accessed on 10 November 2023. Briefly, the quality of the bases was verified. BWA v.0.7.15 (bwa mem) [32] was used to map the reading against the H37Rv genome (NCBI Accession: NC_000962.3), and duplicates were removed using Sambamba software v0.5.0 [33].

### 2.10. Comparative Genomics Analysis

A comparative genomics analysis was performed through the usage of the LASTZ [34] and MAUVE [35] genome alignment tools available as part of Geneious Prime 2023. This analysis was performed using both tools, conducting two global alignments of the 18 genomes isolated from patients, comparing the UT205 (GenBank accession: NC_016934.1, GI: 392384721) with the H37Rv genome (NCBI Accession: NC_000962.3).

### 2.11. Ethical Considerations

The study protocol was approved by the Ethics Committee of the HUEM (Protocol 2015-136-003097-2) for the development of this study. The ethical principles of biomedical research were followed in accordance with resolution 008430 of 1993 of the Ministry of Health of Colombia, where it is classified as a risk-free study, taking into account the optimal clinical practices of the Declaration of Helsinki [36]. All participants in this study were informed of the risk it posed and voluntarily signed the form (with the study identifier code PR130-00-051), providing access to the sputum samples from each patient.

## 3. Results

### 3.1. Population Characteristics

Among the 18 individuals included in this study, the predominant proportion was male, corresponding to 11 individuals (61.2%); a range of ages between 17 and 73 years was represented, and the majority (83.5%) came from the municipality of Cúcuta. In addition, five of the participants in this study (27.8%) had a negative result in their smear microscopy test, only two individuals (11.1%) presented a positive HIV test, and in one of the cases (5.6%), resistance to rifampicin was found (Table 1).

### 3.2. Annotation and Identity Analysis of Mtb Genomes

For the 20 sequences analyzed that included the 18 *Mtb* isolates from NS and evaluated against the UT205 and the H37Rv strains, a total of 3814 genes were found, of which 48 coded for RNAs, and the rest coded for proteins (CDS). The overall identity was found to be 95.9%, with an average alignment length of 4,411,532 base pairs (bps). A total of 4,277,886 identical sites were identified, representing 96.6% similarity.

In relation to the distance matrix, it was found that all *Mtb* genomes from NS showed high homology with the H37Rv genome, with identity percentages that ranged between 98.70% and 99.56%. The 21088X3-col119, 21088X6-col124, 21088X16-col207, 2108X18-col213, 21088X10-col174, 21088X13-col179, 21088X4-col121, 21088X12-col178, 21088X15-col201, and 21088X7-col132 genomes presented the highest identity values (Figure S1).

On the other hand, the global similarity between genomes from NS and the UT205 genome was 98%, with an average alignment length of 4,411,532 bp and a total of 19 sequences evaluated. In this case, 4,276,412 identical sites were reported, representing 96.5% similarity.

When the homology of the genomes was evaluated individually in comparison with the UT205 and H37Rv genomes, identity percentages that varied between 94.80% and 95.41% were found, as reported in the distance matrix.

### 3.3. Phylogenetic Analysis

**Figure 1:**
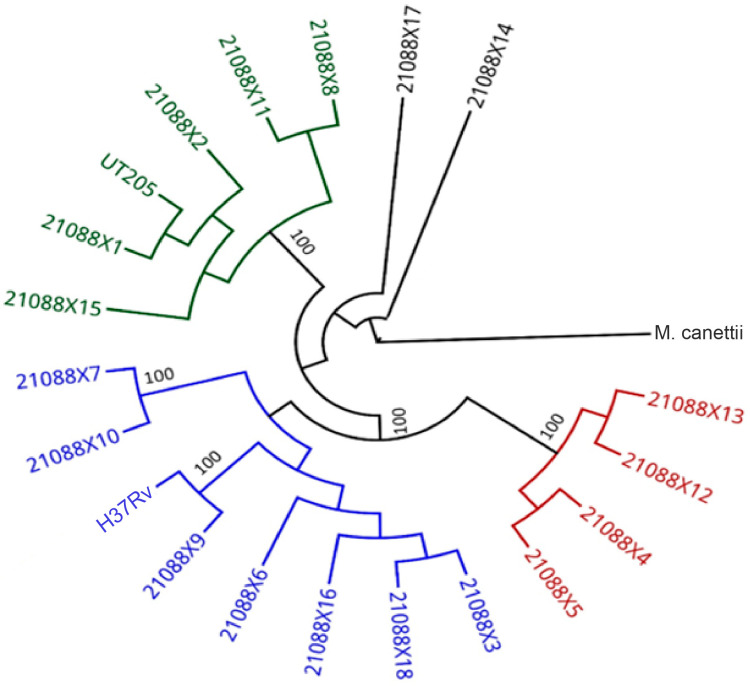
Phylogenetic tree. Phylogeny of the genomes of *Mtb* evaluated against the Colombian UT205 strain and the reference H37Rv genome using the RAxML tool. Group 1 is shown in red, group 2 is shown in blue, and group 3 is shown in green; isolates in black are not related to any group.

The results of the phylogenetic analysis revealed three clusters of *Mtb* isolates, including the UT205 genome, which was compared to the H37Rv genome (Figure 1).

The first group is made up of the genomes of the isolates 21088X13-col179, 21088X12-col178, 21088X4-col121, and 21088X5-col123; the second group includes the H37Rv genome and the isolates 21088X9-col173, 21088X6-col124, 21088X16-col207, 21088X18-col213, 21088X3-col119, 21088X7-col132, and 21088X10-col174; and group three consists of the isolates 21088X15-col201, 21088X1-col41, 21088X2-col117, 21088X11-col177, and 21088X8-col137 and the UT205 genome. Isolates 21088X17-col210 and 21088X14-col180 are not related to any group within the phylogenetic tree.

These results show a grouping according to the homology between genomes. The isolates of group 2 present a greater similarity to the H37Rv genome, while the isolates of group 3 present a greater similarity to the genome of the UT205 strain.

### 3.4. Identification of Mtb Variants

**Figure 2:**
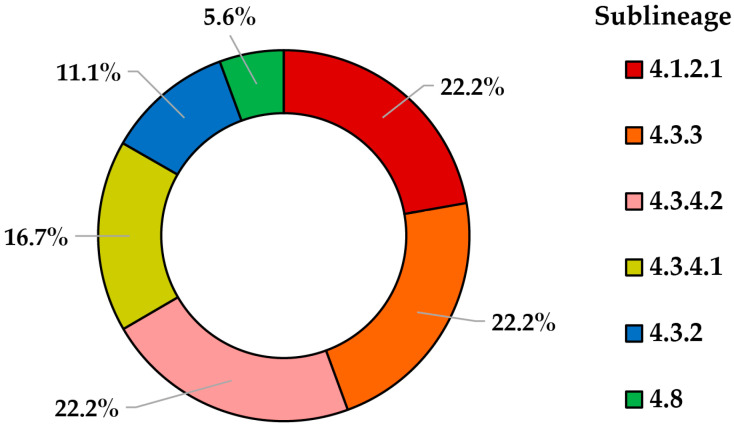
L4 Sublineages of *Mtb* circulating in the municipalities of NS, Colombia.

The genomes of the 18 sequenced isolates could be included in six L4 sublineages and were distributed as follows: 4.1.2.1 (H1) with four isolates; 4.3.2 (LAM3) with two isolates; 4.3.3 (LAM9) with four isolates; 4.3.4.1 (LAM1) with three isolates, 4.3.4.2 (LAM11) with four isolates, and 4.8 (T) with a single isolate (Figure 2). The most frequent sublineages were 4.1.2.1, 4.3.3, and 4.3.4.2, and each one has four isolates (22.2%).

Of the 11 municipalities in which this study was carried out, 4 contributed *Mtb* isolates to the 18 isolates sequenced, and Cúcuta was the municipality that had the highest number of isolates with 15 (83.5%), including all sublineages, while, in the remaining three municipalities of Chinácota, El Zulia, and Tibú, only the 4.1.2.1 and 4.3.4.2 sublineages were found.

### 3.5. Comparative Genomics Analysis

#### 3.5.1. Comparative Genomics Analysis of *Mtb* Isolates against the H37Rv Genome

The comparative genomics analysis against the H37Rv genome revealed a total of 27,151 genetic modifications, of which 25,200 correspond to single-nucleotide polymorphisms (SNPs); of these, 8707 were synonymous, and 16,493 conferred changes in the amino acid residues involved that could have various consequences for the protein. The other gene modifications found correspond to insertions events and deletions of single nucleotides or tandem repetitions, constituting SNPs that confer a loss of the starting codon and generate a truncation in the protein, affecting its length.

A total of 44 genes involved in this group of gene modifications encode proteins that are responsible for the biosynthesis of phenolic glycolipids and derivatives of p-hydroxybenzoic acid (PGL/p-HBAD), proteins related to virulence and the host’s immune response, integral proteins of the membrane, enzymes and metabolic proteins, and proteins associated with gene regulation and transcription (Table 2).

**Figure 3:**
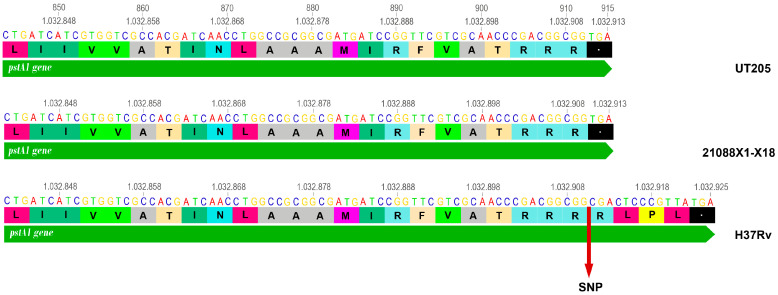
Genetic modification in the gene that codes for the PstA1 protein. In the image, which shows the results generated using LASTZ and Mauve tools, it is observed comparatively against the H37Rv genome a SNP at position 915, a termination codon in the protein causing a loss of 3 amino acids towards the C-terminal region.

The frequencies of the genetic modifications were between 2.9% and 100%, where the proteins PstA1, LpdA, and PE35 represented 100% of the frequency; that is, the variants detected were in all the *Mtb* isolates from NS collected in the present study and in the UT205 strain as well. In the case of the *rv0930* gene that codes for the PstA1 protein, truncation was found in the protein (Figure 3).

In addition, the *rv3303c* and *rv3872* genes coding for the proteins LpdA and PE35, respectively, presented SNPs that conferred truncation in the coding proteins, modifying their extension, as shown in Figures S2 and S3.

**Figure 4:**
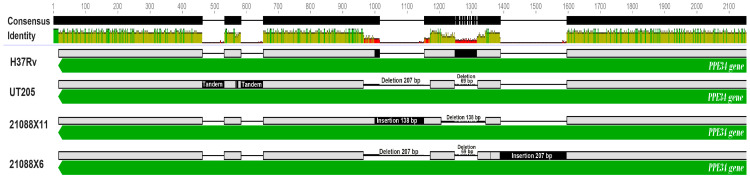
Gene modifications in the gene coding the PPE34 protein. In the image, which shows the results generated using LASTZ and Mauve tools, it is observed comparatively against the H37Rv genome that the UT205 genome is shown, where two tandem insertions of 69 bp were identified, in addition to two deletions of 207 and 69 bp. In the 21088X11 genome, an insertion and a deletion can be observed, both amounting to 138 bp, while, in the 21088X6 genome, two deletions appear, one of 207 bp and the other of 69 bp, as well as an insertion of 207 bp.

The other genes presented gene modifications with lower frequencies affecting specific isolates, for example, the *rv1917c* gene that codes for the PPE34 protein, which has a size of 4366 bp, and in the case of the UT205 genome, two tandem insertions were identified at positions 463 and 583, respectively, that were not in the 18 NS *Mtb* isolates, in addition to two deletions, one of 207 bp (position 964) and the other of 69 bp (position 1248), and a 75 bp insertion (position 2895), as shown in Figure 4.

Similar behavior was observed in the genome of isolate 21088X11-col177, in which a 138 bp insertion at position 1014 and a deletion of the same size at position 1204 were identified (Figure 4), while, in the genome of isolate 21088X6-col124, two of the previously reported deletions in the UT205 strain were identified (207 bp at position 964 and 69 bp at position 1248), in addition to a 207 bp insertion at position 1388 (Figure 4).

#### 3.5.2. Comparative Genomics Analysis of *Mtb* Isolates against to UT205 Genome

The genomic analysis of the 18 isolates from NS against the UT205 genome revealed the presence of various gene variants, identifying a total of 503 variants, of which 196 were located in 123 involved genes. Mutations due to SNPs (141 in total) were the most frequent, of which 57 resulted in synonymous changes, while the 84 SNPs remaining generated modifications in the amino acid residues of related proteins. Furthermore, 37 insertion events were identified, of which 12 involved tandem repeat sequences.

The frequency of the gene variants found was between 22% and 100%, where the maximum value indicates that the variant was presented in the 18 genomes obtained from the isolates from the NS patients analyzed in the present study. In this way, it was determined that 28 variation events presented a frequency of 100% (Table 3), and the genes involved were classified into three main groups according to their biological function in *Mtb.*

**Figure 5:**
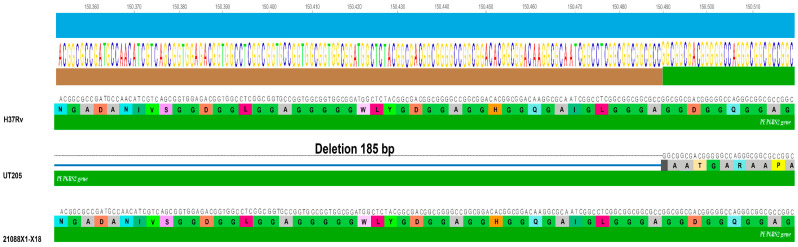
Genetic modifications (SNPs) in the *PE_PGRS2* gene. The image, showing the results obtained using LASTZ and Mauve tools, depicts a deletion in the UT205 genome.

The first group encompasses genes that are related to the coding of PE/PPE family proteins (*PPE34*, *PPE8*, *PPE15*, *PPE59*, *PE_PGRS5*, *PE_PGRS42*, *PE_PGRS59*, *PE_PGRS60*, and *PE_PGRS51*), as described in Table 3, where the *rv1917c* gene that codes for the PPE34 protein is highlighted again, presenting two deletions of 69 nucleotides towards the 3′ region and three insertions of tandem repetitions (two towards the 3′ region and one in the middle region of the sequence) involving a frameshift of the protein, leading to a significant alteration in the sequence of the amino acid and therefore possibly significantly impacting the function of the corresponding protein. In the same way, the *PE_PGRS2* gene presents a genetic modification in UT205 that includes a 185 bp deletion, which generates a significant change in the reading frame of the protein (Figure 5).

The second group of genes includes those associated with lipid biosynthesis and the stress response of mycobacteria. The *sigJ* and *sigM* genes are highlighted, which present a SNP and a short tandem insertion, respectively.

Finally, the third group of genes that presented modifications in all the genomes evaluated consisted of genes related to lipid and carbohydrate metabolism (*aldA*, *rocA*, *cyp144*, *pykA*, *gnd1*, and *glpQ1)*. These are genes that conferred the change in the amino acid residue involved, except for the gnd1 gene, which presented a 5 bp insertion towards the 3′ region, and the *glpQ1* gene, which had a deletion of the same size.

The other genes are associated with various biological processes in *Mtb*, such as the synthesis and metabolism of lipids and fatty acids (*plsB1*, *pks1*, *pks5*, *pks7*, *pks9*, *fadB3*, *fadE28*, and *fadD35*) which frequency remained in the range of 27.8% to 94.4% and wherein the representative gene changes were simple-nucleotide substitutions that conferred a change in the amino acid residues involved.

**Figure 6:**
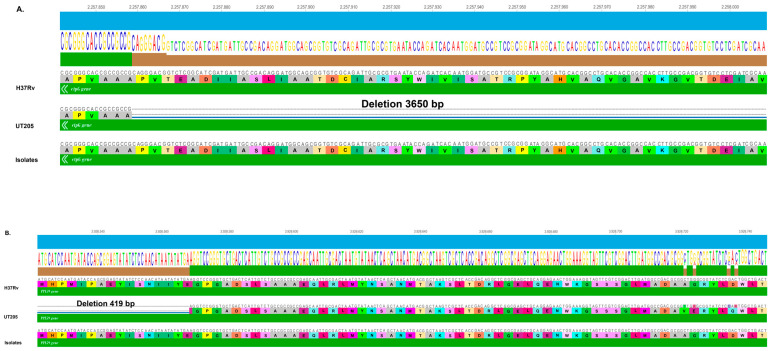
Gene modifications in the *ctpG* and *PPE59* genes. The image, showing results obtained using LASTZ and Mauve tools, shows the following: (**A**) gene deletion of 3650 bp in the *ctpG* gene; (**B**) deletion of 419 bp in the *PPE59* gene present in the UT205 genome.

Other genes that draw attention are *ctpG* and *PPE59*, because they presented deletions of 3650 bp and 419 bp, respectively, in the UT205 strain, which generated a truncation in the coding protein (Figure 6).

## 4. Discussion

This research is the first study carried out in NS, Colombia, in which WGS was used to analyze TB cases. The results allowed the identification of *Mtb* isolates with an orphan genotype (based on *spoligotyping*) in L4 or Euro-American lineages with sublineages 4.1.2.1, 4.3.2, 4.3.3, 4.3.4.1, 4.3.4.2, and 4.8. These results obtained are similar to those reported in previous studies conducted using WGS [38,39] and traditional methods found in the rest of the country [40,41] and the Latin American continent [42].

In the gene identity analysis, it is notable that the 18 isolates present greater homology to H37Rv than UT205. On the other hand, the results of the phylogenetic analysis allowed us to detect three clusters among the *Mtb* isolates, perceiving some degree of greater similarity with the reference strains. However, we recommend conducting a more robust phylogenetic analysis that allows the use of other tools (PhyML and FastTree) and involves various genomic regions that allow the acquisition of precise and efficient phylogenetic reconstructions, which will contribute significantly to the understanding of the evolutionary relationships between microorganisms at the genomic level.

In the sociodemographic characterization of the 18 sequenced isolates obtained from the participants, it was found that all those in sublineage 4.1.2.1 were female, while all were male in sublineages 4.3.4.1, 4.3.4.2, and 4.8. At the same time, in sublineages 4.3.2 and 4.3.3, the participants were equally distributed according to sex. These data show that neither of the two sexes presented exclusivity in terms of carrying a sublineage in this group.

Regarding the relationship between sublineages and the municipalities of origin of the participants, it was found that sublineage 4.1.2.1 corresponded to participants from communes 3, 4, and 6 of Cúcuta, as well as from the municipality of El Zulia; sublineage 4.3.3 corresponded to participants from communes 1, 3, 5, and 6; sublineage 4.3.4.2 corresponded to participants from communes 3, 9, and the municipalities of Chinácota and Tibú; sublineage 4.3.4.1 corresponded to participants from communes 2, 6, and the township of Aguaclara; sublineage 4.3.2 corresponded to participants from commune 9 and the township of Buena Esperanza; and the sublineage 4.8 corresponded to participants from commune 7. The results obtained indicate that the transmission of this disease is not determined by a specific sublineage, since these were found to be indiscriminately present in high- or low-risk areas of the department of NS.

On the other hand, the presence of sublineages 4.3.3, 4.3.4.1, and 4.3.2 was found without distinction in population groups such as homeless and HIV-positive and migrants, being consistent with the findings of classical epidemiology [22], which highlights these population groups as important, because they can become spreaders of the infection in the community, and therefore, it is necessary to prioritize control measures to increase early detection both in studies of contacts and the general population that allow a rapid and timely diagnosis.

Another important finding was that 3 of the 18 participants had negative smear microscopy results in sublineages 4.1.2.1, 4.3.3, and 4.3.4.2. It is important to mention that people with negative bacilloscopy results can transmit TB and thus constitute a hidden prevalence of the disease and promote the maintenance of the chain of transmission in a community [43].

In regard to the grouping of the isolates, sublineages 4.1.2.1, 4.3.2, and 4.3.3 presented grouping in half of their isolates, while sublineages 4.3.4.1 and 4.3.4.2 showed grouping in a lower proportion (25%). However, these results show that five of the six sublineages identified in this study are associated with the recent transmission of TB in Cúcuta and that the six sublineages are related to relapses and reinfections both in the communes of Cúcuta and neighboring municipalities.

Nevertheless, only 1 of the 18 samples was observed to be resistant to rifampicin, corroborating the fact that rifampicin resistance does not constitute a cause for concern in the region [44].

As consequence of the different genetic modifications observed when performing the comparative genomics analysis of the 18 isolates of NS *Mtb* and UT205 against the H37Rv genome, changes in biological function were inferred, such as those generated in a critical site of the protein, potentially negatively or positively affecting the metabolic pathways associated with it, as well as protein–protein interactions and enzymatic activity, among others, which could be associated with adaptation and survival events within the host, as well as evasion of the immune system and susceptibility to drugs [45].

One of the gene modifications found in the NS *Mtb* isolates involves the gene encoding the PstA1 protein that is associated with the high-affinity phosphate transport system in *Mtb*. This phosphate transport system called Pst (“phosphate-specific transport”) is essential for the efficient uptake and utilization of phosphate, a nutrient essential for the growth and survival of bacteria [46]. In the Pst system, PstA1 functions as a periplasmic protein that contributes to the adaptation and survival of mycobacteria in environments where phosphate is a limited resource [46]. It has been shown that the Pi uptake system component of *Mtb*, PstA1, is essential to counteract the IFNγ-dependent immune response [46], and its deficiency could trigger high sensitization of the mycobacteria to the host immune response [46,47,48].

Another modification found was observed in the *rv3303c* gene, which encodes the enzyme lipoamide dehydrogenase (LpdA), which performs an important role in the metabolism (specifically in regard to the generation of energy under anaerobic conditions [49]) and in biosynthesis of the mycolic acids, key components of its cell wall; this modification could be associated with mycobacteria´s resistance against reactive metabolites of nitrogen and nitric oxide, as this enzyme plays an important role in this process [50].

Likewise, the *rv3872* gene, which encodes the PE35 protein belonging to the PE/PPE family, presented a SNP at position 295 that caused the loss in an amino acid at the external C-terminal, producing a truncation of the expression of the corresponding information. As it is a protein associated with the virulence of the microorganism, it could be thought that this modification is a function of the evasion of the immune system; however, more specific studies are required to support this hypothesis. In a study carried out by Jiang and colleagues, among 161 isolates of *Mtb*, mutations in the PE35 protein that generated drastic changes resulting in the increased virulence of the mycobacteria were identified [51].

Additionally, SNPs were found in 2 of the 18 isolates (21088X06-col124 and 21088X11-col177), and they were related to the *rv1917c* gene, which encodes the PPE34 protein, which is associated with the virulence and evasion of the host immune system [52]. This protein helps dendritic cells mature by inducing a Th2 cellular response that favors immune evasion [53]; in addition, together with the rest of the PE/PPE proteins, it exhibits high genetic variability within *Mtb* isolates that may contribute to the adaptation of the bacteria to different environments and immunological conditions, facilitating its survival and persistence [54].

The behavior of the genetic modifications found in this gene in the NS isolates and the UT205 genome could be associated with strain-specific variability, where factors such as a patient’s immunity could influence such changes. Another phenomenon that could be associated is the geographical distribution of the isolates involved in this study (in the NS region) and the Colombian UT205 genome (Medellin) significantly influencing the genetic variability in the gene coding for PPE34. Therefore, it is necessary to highlight that research on PE/PPE proteins continues to shed light on their specific functions and contribution to *Mtb* pathogenesis. A detailed understanding of the biology of these proteins may have important implications for the development of therapeutic strategies and vaccines against TB [52,55].

Similarly, modifications in the proteins associated with the biosynthesis of phenolic products of PGL/p-HBAD were identified in the other genes, which presented frequencies between 11% and 38%, wherein truncation and extension events of the proteins were observed. These changes could be related to the adaptation of mycobacteria to different environments, drug resistance, and interaction with the immune system.

The phenolic products of PGL/p-HBAD in *Mtb* play an essential role in the structure and virulence of the corresponding bacterium, because they form crucial components of the cell wall, contributing to its integrity and resistance [56]. Also, their presence allows modulating the immune response and the ability to evade the host’s defenses. Furthermore, they can contribute to the adaptation of bacteria to different environments within the host, playing a crucial role in the pathogenesis of TB [56,57].

As described, most of the modifications occurred in genes associated with the PE/PPE protein family, presenting frequencies between 2% and 100% (Table 2). These proteins are known for their diversity in sequence and structure, as well as for their role in the interaction with the host immune system and in their contribution to bacterial virulence [52]. The adaptation of *Mtb* to different human populations and geographic environments may be reflected in specific patterns of genetic variability in PE/PEE proteins, suggesting an evolutionary response to host selective pressures and providing deep insight into the complexity of the interaction between this bacterium and the immune system, as well as its ability to persist and cause disease in various populations and epidemiological contexts [54,55,58,59].

Regarding the frequency of occurrence, between 5% and 47% (Table 2) of the modifications present in the genes that encode proteins that interact with the host’s immune response play a role in selection pressure events that could be influenced by the host or environmental factors.

Finally, some variants were identified in genes encoding integral membrane proteins. The frequencies of occurrence are between 5% and 72%, and their possible effects on mycobacteria are associated with resistance or evasion of the immune system due to truncations in these proteins, which could have significant implications for the pathogenesis of TB. These mutations could allow these mycobacteria to avoid recognition by and the effective response of the immune system, thus facilitating their survival and persistence in the host. By avoiding the defenses of the immune system, *Mtb* can establish chronic and recurrent infections by evading immunological and therapeutic interventions. Furthermore, immunological resistance could have consequences for the transmissibility of the disease, affecting the spread and epidemiological burden of TB in affected populations [60].

On the other hand, regarding the gene variants found in the *Mtb* isolates in this study, when performing the comparative analysis against the UT205 genome, it is important to highlight the first group of genes related to the PE/PPE family due to their importance in pathogenesis and in the interaction between the pathogen and the host’s immune system. The literature suggests that this group of proteins could be involved in the evasion of the immune system, affecting the antigen presentation and playing a role in the adaptation of Mtb to the intracellular environment [61]. Similarly, in the *PE_PGRS2* gene, there is a genetic modification in the Colombian UT205 strain, not found in the NS isolates, and its effect seems to be similar to those effects found in the PPE34 protein, which were related to the interaction between the host immune system and *Mtb* [62,63].

The second group, which involves mutations in genes that code for factors called “sigma” involved in gene expression and response to stress, may have significant consequences on the ability of the mycobacteria to adapt to their environment. These “sigma” factors play a key role in the regulation of genes related to virulence and response to adverse conditions [64]. A change could alter the expression of genes critical for the survival and persistence of *Mtb*, compromising its ability to evade the host immune system and resist hostile conditions. This could influence the virulence of the bacteria and their ability to establish chronic infections, thus affecting the dynamics of TB. Furthermore, such a change could have implications for drug resistance and the effectiveness of therapeutic strategies, highlighting the importance of understanding at a molecular level how these changes affect the adaptation of mycobacteria to their changing environment [64,65].

In the third group, gene modifications were found in all 18 isolates related to lipid and carbohydrate metabolism. Mutations in these genes could have various consequences that impact the adaptation of mycobacteria to their environment. For example, SNPs in molecules such as aldolase (*aldA*) and regulatory protease (*rocA)*, which are involved in central metabolism and protein regulation, respectively [66], could affect the ability of mycobacteria to utilize carbon sources and nitrogen. Similarly, mutations in *cyp144* (cytochrome P450) could influence the resistance to xenobiotic compounds and the modulation of virulence. On the whole, mutations in these genes could alter critical aspects of *Mtb* metabolism, stress response, and virulence, influencing *Mtb*´s ability to adapt and persist in its host.

Likewise, other mutations related to the degradation, synthesis, and transport of fatty acids were found (the *plsB1*, *pks1*, *pks5*, *pks7*, *pks9*, *fadB3*, *fadE28*, and *fadD3* genes), which could alter the ability of the bacteria in question to use and metabolize fatty acids, essential components for their survival and persistence in the host [67]. Such mutations could impact lipid biosynthesis, affecting the integrity of the cell wall and, therefore, the bacteria’s resistance to environmental conditions and the host’s immune system. Since lipid homeostasis is essential for *Mtb* virulence, mutations in *fadB3*, *fadE28*, and *fadD35* could influence the analyzed bacteria’s ability to establish chronic infections and evade immune responses, indicating the importance of these genes in the pathogenesis of TB [68].

Finally, it is important to mention that the *ctpG* gene presented an insertion with a frequency of 63.6%, a relatively high value, and was detected in some of the NS isolates in this study (21088X10-col174, 21088X12-col178, 21088X13-col179, 21088X4-col121, 21088X5-col123, 21088X7-col132, and 21088X9-col173). This gene encodes proteins involved in the active transport of metal ions and other cations across the cell membrane, a process with the maintenance of ionic homeostasis and resistance to heavy metals, playing a role in the survival of mycobacteria [69,70].

On the other hand, the gene modification in *PPE59* presented a much lower frequency compared to *ctpG* (36.4%) and was present in only four of the clinical isolates (21088X4-col121, 21088X5-col123, 21088X12-col178, and 21088X18-col213). According to the literature, this gene encodes proteins that are expressed on the cell surfaces of the bacteria in question and may play a role in the evasion of the immune system or in resistance to fluoroquinolones [71].

The genomic comparison of the *Mtb* isolates with the Colombian UT205 strain revealed various genetic modifications that are different from those found when comparing all the isolates with the H37Rv genome. It can be seen that the mutations identified in the comparison with the UT205 genome in all cases correspond to this strain—that is, they are typical of the Colombian strains. The analysis against H37Rv allows to conclude that the majority of the isolates retain more characteristics than the UT205 strain.

This study has limitations regarding the relatively small number of isolates included in the analysis. It is possible that a larger sample may reveal more information about the genetic variations and evolution of *Mtb* strains circulating in the department of NS, Colombia. In addition, future efforts are needed to perform more robust phylogenetic reconstruction analyses to map the genetic diversity present in *Mtb* isolates from the region.

## 5. Conclusions

This is the first description of the molecular epidemiology of *Mtb* isolates circulating in NS achieved through WGS. It was possible to perform a comparative genomics analysis between *Mtb* isolates against the universal references H37Rv and Colombian UT205 genome, which revealed various changes in each genome evaluated that can help us to understand the local genetic diversity and is relevant for epidemiological studies, providing insight into TB transmission dynamics in NS, Colombia.

## Figures and Tables

**Table 1:** Study population characteristics.

Sample	Sex	Age	Municipality of Origen	Commune/Township of Cúcuta	Overcrowding	Homeless	Type of TB	Comorbidity	HIV Result	BK Result *	Drug Susceptibility
col180	M	42	Cúcuta	6	No	HL	Pulmonary	HIV	Positive	2+	Sensitive
col179	M	42	Cúcuta	6	No	HL	Pulmonary	HIV	Positive	3+	Sensitive
col119	F	45	Cúcuta	4	No	No	Pulmonary	None	Negative	Negative	Sensitive
col41	M	68	Cúcuta	Aguaclara	No	No	Pulmonary	None	Negative	2+	Sensitive
col207	F	56	Cúcuta	6	No	No	Pulmonary	None	Negative	Negative	Sensitive
col178	M	32	Cúcuta	5	No	No	Pulmonary	None	Negative	2+	Sensitive
col137	M	71	Cúcuta	3	No	No	Pulmonary	DM	Negative	1+	Sensitive
col177	M	73	Chinácota	Without commune	No	No	Pulmonary	None	Negative	1+	Sensitive
col117	M	26	Cúcuta	9	No	No	Pulmonary	None	Negative	Negative	Sensitive
col213	F	17	El Zulia	Without commune	No	No	Pulmonary	None	Negative	3+	Sensitive
col201	M	72	Tibú	Without commune	No	No	Pulmonary	None	Negative	1+	Sensitive
col174	F	28	Cúcuta	Buena Esperanza	No	No	Pulmonary	Malnutrition	Negative	1+	Sensitive
col173	M	51	Cúcuta	7	No	No	Pulmonary	Malnutrition	Negative	1+	Sensitive
col132	M	71	Cúcuta	9	No	HL	Pulmonary	Malnutrition	Negative	3+	Sensitive
col124	F	33	Cúcuta	3	No	No	Pulmonary	None	Negative	Negative	Sensitive
col123	F	33	Cúcuta	3	No	No	Pulmonary	None	Negative	Negative	Sensitive
col210	M	32	Cúcuta	2	No	No	Extrapulmonary	None	Negative	Without Data	RR
col121	F	22	Cúcuta	1	No	No	Pulmonary	None	Negative	3+	Sensitive

Note. HL: homeless, DM: diabetes mellitus, and RR: rifampicin resistance. * WHO scale: Negative: No AFB found in at least 100 microscopic fields, 1+: 10 to 99 AFB per 100 microscopic fields, 2+: 1 to 10 AFB per field in at least 50 microscopic fields, and 3+: more than 10 AFB per microscopic field in at least 20 fields [37].

**Table 2:** Genetic variants found in *Mtb* isolates compared to the H37Rv genome.

Gene Involved	Protein Coding	Type of Modification	Variant Frequency	Isolates Involved	Protein Effect	Biological Function
*rv2958c*	PGL/p-HBAD biosynthesis glycosyltransferase	Substitutions	11.10%	21088X12, X15	Protein spread	PGL/p-HBAD Biosynthesis
*rv2962c*	PGL/p-HBAD biosynthesis rhamnosyltransferase	SNP Transition	22%	21088X4, X5, X12, X13	Truncation	
*rv2959c*	Rhamnosyl O-methyltransferase	SNP Transition	38.9%	21088X1, X2, X8, X11, X14, X15, X17	Truncation	
*rv0279c*	PE PGRS4	Substitutions	33.3%	21088X1, X6, X7, X9, X10, X11, X12, X14, X17	Protein spread	Virulence and evasion of the host immune response
*rv0978c*	PE PGRS17	SNP transversion	41.90%	21088X1, X2, X3, X4, X5, X6, X7, X8, X9, X11, X13, X16, X18	Protein spread	
*rv0980c*	PE PGRS18	SNP transversion	21.70%	21088X10, X12, X14, X15, X17	Truncation	
*rv3344c*	PE PGRS49	Substitutions	35.70%	21088X1, X2, X4, X5, X7, X9, X10, X12, X13, X14	Loss of start codon	
*rv3508*	PE PGRS54	Substitutions	5.3%	21088X5	Protein spread	
			23.50%	21088X1, X2, X3, X6, X12, X13, X15, X18	Truncation	
*rv0304c*	PPE5	Substitutions	50%	21088X2, X3, X4, X5, X6, X7, X8, X9, X10, X11, X12, X13, X14, X15, X16, X17, X18	Loss of start codon	
*rv0305c*	PPE6	Substitutions	2.9%	21088X11	Truncation	
			45.70%	21088X2, X3, X4, X5, X6, X7, X8, X9, X10, X12, X13, X14, X15, X16, X17, X18	Protein spread	
*rv0354c*	PPE7	SNP transversion	47.10%	21088X2, X3, X4, X5, X6, X7, X8, X9, X10, X12, X13, X14, X15, X16, X17, X18	Protein spread	
*rv1917c*	PPE34	SNP	8.20%	21088X12, X13, X17	Protein spread	
*rv3125c*	PPE49	SNP transversion	22.20%	21088X3, X6, X16, X18	Truncation	
*rv3872*	PE35	SNP transversion	100%	21088X1-18	Truncation	
*rv0180c*	Transmembrane protein	SNP Transition	5.60%	21088X9	Truncation	Membrane proteins
*rv0446c*	Transmembrane protein	SNP Transition	72%	21088X1, X2, X4, X5, X7, X8, X10, X11, X12, X13, X14, X15, X17	Truncation	
*rv1624c*	Integral membrane protein	SNP Transition	11.10%	21088X4, X5	Truncation	
*rv2120c*	Integral membrane protein	SNP Transition	11.10%	21088X10, X11	Truncation	
*rv2395*	Membrane protein	SNP Transition	11.10%	21088X12, X13	Truncation	
*rv3870*	EccCa1 CDS	Substitutions	35.70%	21088X3, X5, X6, X7, X8, X9, X12, X14, X16, X17	Protein spread	Proteins related to the immune response
*rv0305c*	PPE6 CDS	Substitutions	2.90%	21088X11	Truncation	
			45%	21088X2, X3, X4, X5, X6, X7, X8, X9, X10, X12, X13, X14, X15, X16, X17, X18	Protein spread	
*rv0354c*	PPE7 CDS	SNP Transition	47.10%	21088X2, X3, X4, X5, X6, X7, X8, X9, X10, X12, X13, X14, X15, X16, X17, X18	Extension	
*rv0577*	TB27.3	SNP transversion	5.60%	21088X11	Truncation	
*rv0113*	GmhA CDS	SNP transversion	5.90%	21088X2	Truncation	
*rv2221c*	GlnE CDS	SNP Transition	5.60%	21088X9	Loss of start codon	
*rv2673*	AftC CDS	SNP Transition	33.30%	21088X3, X4, X7, X8, X9, X12, X13, X14, X18	Truncation	
*rv0197*	Oxidoreductase	SNP transversion	88.90%	21088X1, X2, X3, X6, X7, X8, X9, X10, X11, X12, X13, X14, X15, X16, X17, X18	Truncation	Enzymes and metabolic proteins
*rv0389*	PurT CDS	SNP Transition	5.60%	21088X9	Truncation	
*rv0873*	FadE10 CDS	SNP Transversion	5.60%	21088X17	Truncation	
*rv0930*	PstA1 CDS	SNP Transition	100%	21088X1-18	Truncation	
*rv3287c*	RsbW CDS	SNP Transversion	5.60%	21088X17	Truncation	
*rv3303c*	LpdA CDS	SNP Transversion	100%	21088X1-18	Truncation	
*rv3618*	Monooxygenase	SNP Transition	11.10%	21088X4, X5	Truncation	
*rv3378c*	Diterpene synthase	SNP Transversion	11.10%	21088X1, X14	Truncation	
*rv3701c*	EgtD CDS	SNP Transversion	5.60%	21088X9	Truncation	
*rv3097c*	LipY CDS	SNP Transition	5.60%	21088X6	Truncation	
*rv1167c*	Transcriptional regulator	SNP Transversion	5.60%	21088X2	Protein spread	Regulation and transcription proteins
*rv3050c*	Asn C family transcriptional regulator	SNP Transition	5.60%	21088X17	Truncation	
*rv1189*	SigI CDS	SNP Transversion	5.60%	21088X7	Truncation	
*rv1329c*	DinG CDS	SNP Transversion	5.60%	21088X10	Truncation	
*rv1493*	MutB CDS	SNP Transition	47.10%	21088X2, X3, X4, X5, X6, X7, X8, X9, X10, X11, X12, X13, X15, X16, X17, X18	Truncation	
*rv1494*	MazE4 CDS	SNP Transversion	47.10%	21088X2, X3, X4, X5, X6, X7, X8, X9, X10, X11, X12, X13, X15, X16, X17, X18	Truncation	
*rv1574*	Phage protein	SNP Transversion	50%	21088X3, X6, X9, X16, X18	Protein spread	
*rv1575*	Phage protein	SNP Transversion	50%	21088X3, X6, X9, X16, X18	Truncation	

**Table 3:** Variants found in *Mtb* isolates compared to the UT205 genome.

Gene Involved	Protein Coding	Type of Modification	Variant Frequency	Protein Effect	Biological Function
*rv1917c*	PPE34 CDS	Nucleotide insertion	100%	Protein spread	Virulence and evasion of the host immune response
*rv0355c*	PPE8 CDS				
*rv1039c*	PPE15 CDS				
*rv19c*	PPE59 CDS				
*rv0297*	PE PGRS5 CDS				
*rv2487c*	PE PGRS42 CDS				
*rv0376*	PE PGRS59 CDS				
*rv3652*	PE PGRS60 CDS				
*rv356*	PE PGRS51 CDS				
*ppsA*	PpsA CDS	Substitution	100%	No effect	Lipid biosynthesis and stress response
*kdtB*	KdtB CDS	Substitution			
*sigJ*	SigJ CDS	Substitution			
*sigM*	SigM CDS	Tandem deletion		Change in reading frame	
*aldA*	AldA CDS	SNPs	100%	Amino acide residue change No effects of truncation or change in reading frames have been observed	Lipid and carbohydrate metabolism
*rocA*	RocA CDS	SNPs			
*cyp144*	Cyp144 CDS	SNPs			
*pykA*	PykA CDS	SNPs			
*gnd1*	Gnd1 CDS	SNPs			
*gnd1*	Gnd1 CDS	Insertion		Change in reading frame	
*glpQ1*	GlpQ1 CDS	Tandem deletion			
*plsB1*	PlsB1 CDS	SNPs	100%	Amino acide residue change No effects of truncation or change in reading frames have been observed	Lipid and carbohydrate metabolism
*pks1*	Pks1 CDS				
*pks5*	Pks5 CDS				
*pks7*	Pks7 CDS				
*pks9*	Pks9 CDS				
*fadB3*	FadB3 CDS				
*fadE28*	FadE28 CDS				
*fadD35*	FadD35 CDS				

## Data Availability

The raw dataset generated and analyzed during this study will be provided by the authors upon reasonable request.

## Supplementary Materials

The following supporting information can be downloaded at https://www.mdpi.com/article/10.3390/tropicalmed9090197/s1. Figure S1. Identity matrix. The image, corresponding to the results obtained using the LASTZ tool, shows the identity matrix generated for the *Mtb* genomes of this study and the Colombian UT205 strain, and, compared to the universal reference, the H37Rv genome. Figure S2. Gene modification in the gene that codes for the LpdA protein. As revealed using LASTZ and Mauve, the image shows a SNP in position 1416 observed during comparison against the H37Rv genome, where a termination codon is generated in the protein, leading to a loss of 21 amino acids towards the C-terminus. Figure S3. Genetic modification in the gene that codes for the PE35 protein. As revealed using LASTZ and Mauve and in comparison with the H37Rv genome, the image reveals a SNP in position 295, where a termination codon is generated in the protein, leading to a loss of one amino acid towards the C-terminus. Table S1. Summary of the total genome sequencing process of the 18 *Mtb* isolates from NS, Colombia.
