# Supplementary material for: Comparative Genomic Analysis of Mycobacterium tuberculosis Isolates Circulating in North Santander, Colombia
Source: Trop Med Infect Dis. 2024 Aug 28;9(9):197. doi: 10.3390/tropicalmed9090197 (PMC11436241; doi:10.3390/tropicalmed9090197)
Supplement: Supplementary file 1 [file tropicalmed-09-00197-s001.zip › tropicalmed-3156326-supplementary.pdf]

Supplementary material

	H37Rv_M. t...	UT205	21088X3	21088X1	21088X6	21088X9	21088X16	21088X18	21088X15	21088X17	21088X11	21088X10	21088X13	21088X2	21088X8	21088X14	21088X4	21088X12	21088X5	21088X7
H37Rv_M. tuberculosis_		95.36%	99.47%	99.18%	99.53%	99.12%	99.54%	99.64%	99.04%	99.10%	99.04%	99.60%	99.54%	99.08%	99.06%	99.23%	99.55%	99.55%	99.55%	99.60%
UT205	95.36%		95.16%	95.27%	95.10%	94.80%	95.16%	95.11%	95.33%	95.33%	95.33%	95.22%	95.23%	95.36%	95.35%	95.34%	95.23%	95.23%	95.23%	95.27%
21088X3	99.47%	95.16%		98.70%	99.48%	98.80%	99.56%	99.49%	98.73%	98.71%	98.70%	99.15%	99.15%	98.74%	98.73%	98.77%	99.16%	99.15%	99.16%	99.18%
21088X1	99.18%	95.27%	98.70%		98.76%	98.39%	98.76%	98.84%	98.95%	99.02%	98.94%	99.02%	98.95%	98.98%	98.97%	99.16%	98.97%	98.95%	98.97%	99.01%
21088X6	99.53%	95.10%	99.48%	98.76%		98.85%	99.53%	99.47%	98.64%	98.70%	98.62%	99.21%	99.13%	98.66%	98.64%	98.83%	99.14%	99.13%	99.14%	99.18%
21088X9	99.12%	94.80%	98.80%	98.39%	98.85%		98.86%	98.78%	98.27%	98.32%	98.25%	98.80%	98.73%	98.29%	98.28%	98.45%	98.73%	98.73%	98.73%	98.76%
21088X16	99.54%	95.16%	99.56%	98.76%	99.53%	98.86%		99.55%	98.72%	98.71%	98.70%	99.21%	99.21%	98.74%	98.72%	98.83%	99.21%	99.22%	99.21%	99.17%
21088X18	99.64%	95.11%	99.49%	98.84%	99.47%	98.78%	99.55%		98.80%	98.79%	98.78%	99.29%	99.29%	98.82%	98.81%	98.91%	99.30%	99.30%	99.30%	99.27%
21088X15	99.04%	95.33%	98.73%	98.95%	98.64%	98.27%	98.72%	98.80%		99.01%	99.01%	98.90%	98.91%	99.06%	99.04%	99.02%	98.92%	98.91%	98.92%	98.94%
21088X17	99.10%	95.33%	98.71%	99.02%	98.70%	98.32%	98.71%	98.79%	99.01%		98.99%	98.96%	98.90%	99.03%	99.01%	99.11%	98.90%	98.90%	98.90%	99.00%
21088X11	99.04%	95.33%	98.70%	98.94%	98.62%	98.25%	98.70%	98.78%	99.01%	98.99%		98.89%	98.90%	99.05%	99.05%	99.00%	98.90%	98.90%	98.90%	98.93%
21088X10	99.60%	95.22%	99.15%	99.02%	99.21%	98.80%	99.21%	99.29%	98.90%	98.96%	98.89%		99.38%	98.92%	98.91%	99.08%	99.39%	99.39%	99.39%	99.46%
21088X13	99.54%	95.23%	99.15%	98.95%	99.13%	98.73%	99.21%	99.29%	98.91%	98.90%	98.90%	99.38%		98.93%	98.92%	99.03%	99.49%	99.56%	99.48%	99.37%
21088X2	99.08%	95.36%	98.74%	98.98%	98.66%	98.29%	98.74%	98.82%	99.06%	99.03%	99.05%	98.92%	98.93%		99.07%	99.04%	98.94%	98.93%	98.94%	98.97%
21088X8	99.06%	95.35%	98.73%	98.97%	98.64%	98.28%	98.72%	98.81%	99.04%	99.01%	99.05%	98.91%	98.92%	99.07%		99.03%	98.93%	98.92%	98.93%	98.95%
21088X14	99.23%	95.34%	98.77%	99.16%	98.83%	98.45%	98.83%	98.91%	99.02%	99.11%	99.00%	99.08%	99.03%	99.04%	99.03%		99.03%	99.03%	99.03%	99.07%
21088X4	99.55%	95.23%	99.16%	98.97%	99.14%	98.73%	99.21%	99.30%	98.92%	98.90%	98.90%	99.39%	99.49%	98.94%	98.93%	99.03%		99.48%	99.57%	99.38%
21088X12	99.55%	95.23%	99.15%	98.95%	99.13%	98.73%	99.22%	99.30%	98.91%	98.90%	98.90%	99.39%	99.56%	98.93%	98.92%	99.03%	99.48%		99.48%	99.37%
21088X5	99.55%	95.23%	99.16%	98.97%	99.14%	98.73%	99.21%	99.30%	98.92%	98.90%	98.90%	99.39%	99.48%	98.94%	98.93%	99.03%	99.57%	99.48%		99.38%
21088X7	99.60%	95.27%	99.18%	99.01%	99.18%	98.76%	99.17%	99.27%	98.94%	99.00%	98.93%	99.46%	99.37%	98.97%	98.95%	99.07%	99.38%	99.37%	99.38%	

Figure S1. Identity matrix. The image, corresponding to the results obtained using the LASTZ tool, shows the identity matrix generated for the *Mtb* genomes of this study and the Colombian UT205 strain, and, compared to the universal reference, the H37Rv genome

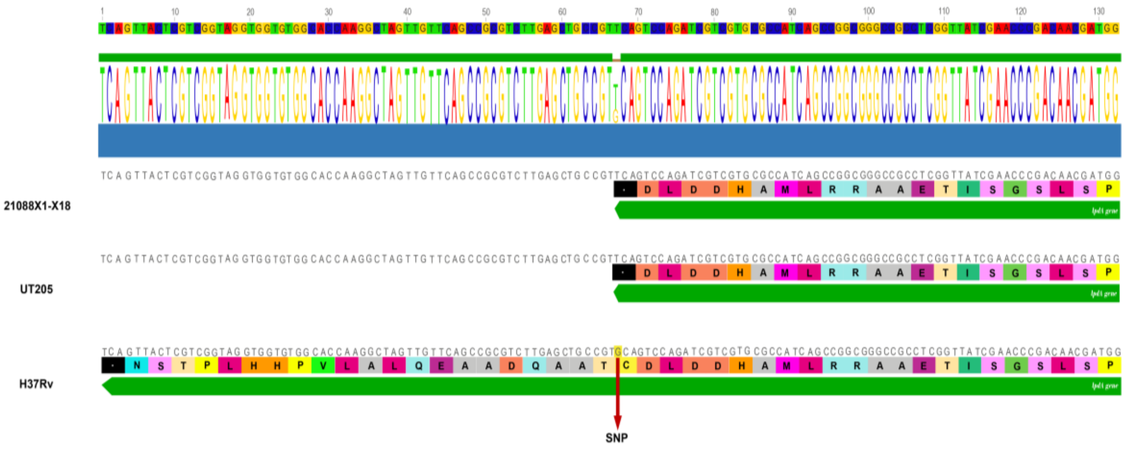

Figure S2. Gene modification in the gene that codes for the LpdA protein. As revealed using LASTZ and Mauve, the image shows a SNP in position 1416 observed during comparison against the H37Rv genome, where a termination codon is generated in the protein, leading to a loss of 21 amino acids towards the C-terminus.

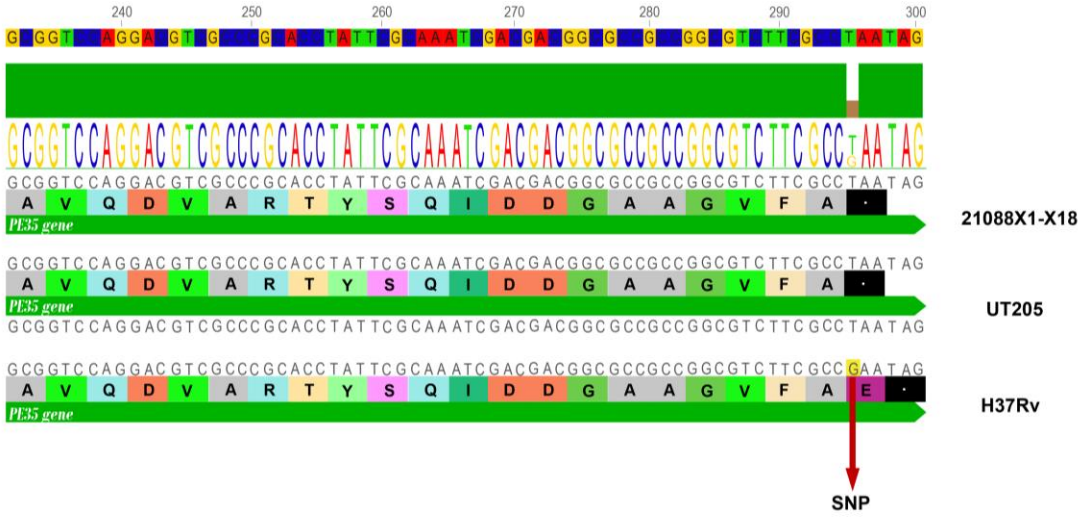

Figure S3. Genetic modification in the gene that codes for the PE35 protein. As revealed using LASTZ and Mauve and in comparison with the H37Rv genome, the image reveals a SNP in position 295, where a termination codon is generated in the protein, leading to a loss of one amino acid towards the C-terminus.

Table S1. Summary of the total genome sequencing process of the 18 *Mtb* isolates from NS,-Colombia

Sample	Raw Readings	Kraken2 Raw Reads	Mapping Percentage	Duplication Percentage	Mean Average	Average Median	Standard Devation Coverage	Strain	Drug Suscetibility
21088X1-col41	11708179	11548446	9901	475811	244174287	2500	36504653	Sublineage 4.3.4.1	Sensitive
21088X10-col174	8137054	7949226	9908	440964	244618997	2500	33695089	Sublineage 4.3.2	Sensitive
21088X11-col177	9016126	8887466	9917	457427	243782422	2500	37602449	Sublineage 4.3.4.2	Sensitive
21088X12-col178	10814160	10598887	9921	458044	245224076	2500	32406841	Sublineage 4.3.3	Sensitive
21088X13-col179	13195879	12858506	9921	502787	245267081	2500	32519089	Sublineage 4.3.3	Sensitive
21088X14-col180	10467736	10233852	9896	453349	244370312	2500	35736174	Sublineage 4.3.4.1	Sensitive
21088X15-col201	10134066	9915945	9918	451118	242262152	2500	397575	Sublineage 4.3.4.2	Sensitive
21088X16-col207	9865634	9665538	9893	454997	245390007	2500	31865414	Sublineage 4.1.2.1	Sensitive
21088X17-col210	9800398	9576085	9857	456434	24393958	2500	37103449	Sublineage 4.3.4.1	RR
21088X18-col213	10711504	10546045	9853	47214	245893371	2500	29984142	Sublineage 4.1.2.1	Sensitive
21088X2-col117	11809835	11629355	9915	466132	243923179	2500	37302744	Sublineage 4.3.4.2	Sensitive
21088X3-col119	12554079	12363025	9916	479819	245637338	2500	31362259	Sublineage 4.1.2.1	Sensitive
21088X4-col121	10342768	10190715	9916	480667	245531307	2500	31166759	Sublineage 4.3.3	Sensitive
21088X5-col123	9261568	9133502	9925	465677	245139714	2500	31962692	Sublineage 4.3.3	Sensitive
21088X6-col124	10399509	10255025	9896	473448	246043353	2500	29505904	Sublineage 4.1.2.1	Sensitive
21088X7-col132	10650540	10493918	9921	490335	246150082	2500	28951075	Sublineage 4.3.2	Sensitive
21088X8-col137	10723950	10553610	9923	462616	244012579	2500	37043381	Sublineage 4.3.4.2	Sensitive
21088X9-col173	2674591	2449927	9921	424609	112594843	1020	60887474	Sublineage 4.8	Sensitive

Note. RR: rifampicin resistance
